# Integrated disability evaluation in low back pain: feasibility and multidomain profiling from the EL DORADO cohort

**DOI:** 10.3389/fresc.2026.1731260

**Published:** 2026-03-18

**Authors:** Anders Hansen, Steen Harsted, Casper Nim, Kieran O’Sullivan, Søren O’Neill

**Affiliations:** 1 Spine Centre of Southern Denmark, University Hospital of Southern Denmark, Kolding, Denmark; 2 Department of Regional Health Research, University of Southern Denmark, Odense, Denmark; 3 Department of Sports Science and Biomechanics, University of Southern Denmark, Odense, Denmark; 4 SDU Health Informatics and Technology, The Maersk Mc-Kinney Moller Institute, Odense, Denmark; 5 School of Allied Health, University of Limerick, Limerick, Ireland; 6 Ageing Research Centre, Health Research Institute, University of Limerick, Limerick, Ireland; 7 Sports and Human Performance Research Centre, Health Research Institute, University of Limerick, Limerick, Ireland

**Keywords:** disability evaluation, electronic health records, low back pain, motion analysis, patient-reported outcomes, performance-based assessment, quantitative sensory testing

## Abstract

Disability evaluation in low back pain (LBP) has traditionally relied on patient-reported outcomes (PROs), which alone incompletely capture functioning. This study tested the feasibility of embedding a multidomain disability evaluation protocol into routine secondary care, integrating PROs with performance-based measures, kinematic analysis, quantitative sensory testing (QST), and electronic health record (EHR) data, without performing formal multidomain profiling or subgroup identification. In this proof-of-concept study, 542 patients referred for advanced LBP assessment at the Spine Center of Southern Denmark underwent multidomain evaluation, including PROs (ODI, EQ-5D-5L, NPRS, STarT Back), functional capacity tests, markerless kinematic motion capture, QST, and EHR extraction; a subgroup provided 60-week SMS follow-up. Feasibility outcomes, including workflow integration, data completeness, acceptability, and adverse events, were evaluated overall and after the first 100 participants to guide protocol refinement. The assessment battery was completed in a mean of 22 min with over 95% data completeness, high acceptability, acceptable patient burden, and only two minor adverse events. PROs indicated moderate disability (ODI 34/100) and poor health (EQ-5D VAS 55/100), while functional and kinematic measures revealed substantial impairments, and QST showed widespread pain sensitivity with minimal conditioned pain modulation. EHR linkage was achieved for all participants, with MRI data available in 88%, and SMS follow-up retention was 75% at 60 weeks. These findings demonstrate that integrating PROs, performance-based tests, QST, kinematic assessment, and EHR data into routine secondary care is feasible, safe, and acceptable. The resulting multidomain dataset provides an infrastructure for future research on prognosis, subgrouping, and disability evaluation in LBP.

## Introduction

Low back pain (LBP) is the leading cause of disability worldwide and accounts for more years lived with disability than any other condition. Its physical, psychological, and social consequences impose substantial societal and economic costs (1, 2). Standard diagnostic triage distinguishes between specific spinal pathology, radicular syndrome, and non-specific LBP (3), but provides limited prognostic precision, as existing prediction models have shown variable performance and limited clinical uptake in individualized rehabilitation or disability-related decision-making (4). Although functional capacity can be measured objectively, the heterogeneity of LBP and the absence of clinically meaningful prognostic classifications make it difficult to judge stability or potential for improvement (4). As a result, disability evaluation remains challenging, particularly in complex cases, and is often based on incomplete representations of functioning rather than integrated assessments.

Disability evaluation in LBP has traditionally relied on patient-reported outcome measures (PROs) such as the Oswestry Disability Index (ODI) (5) and Roland–Morris Disability Questionnaire (RMDQ) (6). Although robust and widely used, these instruments were developed more than three decades ago, before the biopsychosocial model and the International Classification of Functioning, Disability and Health (ICF) became central components in rehabilitation. Consequently, they primarily assess activity limitation related to pain and physical function, with limited coverage of participation, contextual, and psychosocial factors directly relevant to long-term disability (7). Reliance on such a single-domain self-report may therefore inadequately reflect the multidimensional nature of disability in LBP.

The ICF conceptualizes disability as multidimensional, arising from interactions between body impairments, activity limitations, and participation restrictions, all shaped by personal and environmental factors (8). A comprehensive evaluation should therefore integrate multiple complementary data sources, each reflecting a distinct aspect of functioning. PROs capture the lived experience of pain, psychological distress, and perceived participation restrictions. Performance-based functional capacity and kinematic assessments provide observable activity-level functioning under standardized conditions. Quantitative sensory testing (QST) characterizes impairments in pain processing and modulation, linking body functions to activity limitations. Electronic health record (EHR) data adds contextual information on diagnoses, imaging findings, comorbidities, and sickness absence, situating the functional profile within the broader clinical trajectory. Each domain has inherent limitations. Self-report may be information-biased, whereas performance tests may not reflect real-life ecological demands (9). QST characterizes impairment but not participation, while EHR data are influenced by documentation practices and coding variability (10). Such limitations underscore the need for careful standardization and transparent reporting when integrating multiple domains, rather than reliance on any single source of information.

Integrated, multidomain assessment frameworks have therefore been proposed as a means of more comprehensively characterizing functioning in LBP, consistent with the ICF model (7). The EL DORADO study was designed in response to this conceptual need by systematically combining PROs, performance-based measures, bedside QST, and EHR data in a large cohort of patients undergoing advanced diagnostics for LBP. By embedding these assessments directly into routine secondary care at an outpatient hospital unit specializing in spinal care, the EL DORADO captures a population with complex and persistent symptoms, providing detailed multidomain data not typically available in population-based cohorts such as LB^3^P (11). It aims to evaluate the feasibility of integrating a comprehensive, multidomain assessment of functioning into routine secondary care, focusing on workflow integration, data completeness, acceptability, and safety. A secondary objective was to establish a harmonized dataset spanning PROs, performance-based measures, QST, kinematic assessment, and EHR data to support future research on prognosis, subgrouping, and disability evaluation.

## Methods

### Study design

This observational proof-of-concept feasibility study was conducted at the Spine Center of Southern Denmark, which evaluates patients with persistent or complex LBP unresponsive to primary care in the Region of Southern Denmark (population of 1.23 million) (12).

The Regional Committee on Health Research Ethics (S-20220084) granted ethical approval, and all participants provided informed consent.

### Eligibility criteria and recruitment

#### Eligibility criteria

Participants were eligible for inclusion if they were:

1. aged 18 years or older,
2. spoke Danish,
3. referred to the Spine Center of Southern Denmark for secondary care assessment of LBP with or without radiculopathy,
4. able to provide informed consent and participate in the physical assessment procedures. Patients were excluded if they had suspected or confirmed spinal pathology (e.g., fracture, infection, malignancy), required acute spinal surgery, or were unable to complete the assessment procedures safely.

#### Recruitment

Patients were invited via their appointment letter or by the treating clinician following the consultation. Because recruitment was embedded within routine clinical workflows, no independent screening log of all eligible patients was available, and the total eligible population and inclusion rate could not be determined.

### Data sources and management

Data were collected across four complementary domains to provide a multidimensional profile of functioning: (1) PROs; (2) performance-based functional capacity and kinematic assessments; (3) QST; and (4) EHR data for diagnostic, imaging, and care trajectory context. No composite scores, weighting, or formal multidomain integration was performed as part of the present analyses.

### Patient-reported outcomes

Baseline PRO data were retrieved through the *SpineData registry* (13) and completed by participants as part of their initial clinical assessment. These included validated instruments such as the Oswestry Disability Index (ODI) (5), EuroQol (EQ-5D-5L) (14), STarT Back Screening Tool (SBST) (15), and Numeric Pain Rating Scale (NPRS) (16). Additional single-item questions captured sociodemographic variables (age, sex, education) and lifestyle factors (e.g., smoking, physical activity, body mass index). Categorization was conducted according to established guidelines, and missing items were addressed in accordance with published recommendations where available.

Participants also completed a standardized digital pain drawing using a computer mouse or their finger on a touchscreen to draw pain areas on a body chart. Pain locations were recorded as vector data in a Cartesian coordinate system. These vector-based drawings provide spatial representations of symptom distribution, enable the quantification of pain extent, and facilitate location-specific analyses.

The SpineData registry typically required 15–20 min to complete and was administered no more than 7 d before, during, or immediately after the EL DORADO assessment.

### Text message follow-up

A subgroup consented to provide weekly SMS-based spinal pain intensity ratings on a 0–10 scale for 60 weeks. A single automated reminder was sent 24 h later to enhance compliance if no response was recorded. At weeks 15 and 60, NPRS and ODI were also assessed (ODI data not presented here).

### Performance-based measures

Trained clinicians conducted performance-based assessments using standardized protocols immediately before or after the clinical baseline consultation.

### Functional capacity assessments

Functional capacity was assessed through standardized physical performance and capacity tests, including:

- 2-Minute Walk Test (2MWT): Assessed walking capacity along a 30-m indoor corridor. The test was timed with a stopwatch, and assessors recorded the number of completed 30-m laps. At the two-minute mark, the remaining distance on the final lap was measured to the nearest 0.1 m using a laser range finder (DeWalt DW033), and the total distance was calculated in meters (12).
- Five-Repetition Sit-to-Stand Test (5xSTS): Evaluated lower-limb strength and transitional movement. Participants rose from and returned to a standardized chair (seat height 0.48 m) five times as quickly as possible, with or without arm support. Completion time was recorded in seconds (17), and participants who could not complete a pretrial were excluded.
- Hand Grip Strength: Measured using a calibrated dynamometer (Baseline BIMS Digital Grip Dynamometer) following standard testing procedures. Two maximal efforts were recorded, and the mean was reported in kilograms (18).

### Kinematic assessments

Markerless motion capture was performed using four synchronized Razer Kiyo Pro cameras (60 fps) and the open-source SkellyCam platform. 3D pose estimation was conducted with CapturyStudio (The Captury GmbH, Saarbrücken, Germany) (19). All systems were calibrated before each session. Kinematic assessments were designed to capture movement quality and postural stability, providing complementary information to functional capacity tests.

Participants performed standardized tasks with instruction and demonstration. Recorded tasks included:

- Postural Sway: Quiet barefoot standing with feet shoulder-width apart for up to 30 s. Center-of-mass trajectory and sway area were extracted to quantify static balance.
- Spinal Range of Motion: Maximal flexion-extension and lateral flexion from standing.
- Pencil Pick-Up: Reaching for a pencil placed 30 cm in front of the feet using the patient's preferred movement strategy.
- Lifting Task: Participants lifted a 6-kg kettlebell from the floor to a hip-height table using their preferred movement strategy.
- 5xSTS: The test described above was performed with concomitant motion capture recording.
- Treadmill Walking: Participants walked at a 0% gradient. Before testing, they completed up to 1 min of familiarization, ending it earlier if they were comfortable. The initial speed was set to 3 km/h to account for treadmill acceleration, after which participants adjusted the speed as desired using only the up/down controls; all other display information was concealed. Steady-state walking was recorded for at least 30 s, and participants with balance impairments were allowed to use a bar handle for support.

### Quantitative sensory testing

QST was preceded by standardized instructions:

- Pressure Pain Threshold (PPT): Measured at two sites; (i) 5 cm laterally to the L4 segment and (ii) over the muscle belly of the tibialis anterior using a handheld algometer (1.5 cm^2^ probe) at ∼1 kg/s. The mean of two trials was recorded per site on the dominant-hand side. Testing was discontinued at 10 kg if no pain was reported (20).
- Cold Pressor Test: Participants immersed their non-dominant hand in 1 ± 0.3 °C water for up to 120 s or until intolerable. Pain intensity was continuously rated using an electronic visual analogue scale (eVAS) at 1 Hz, capturing duration, VASmax, time to VASmax, and area under the curve (VAS-AUC).
- Temporal Summation of Pain: Assessed before and immediately after the cold pressor using 10 repeated pressure stimuli to the tibialis anterior at 1 Hz. Pain ratings after the 1st and 10th stimuli were recorded (21).
- Conditioned Pain Modulation (CPM): Evaluated by comparing PPT and temporal summation before and after cold pressor exposure.

### Electronic health record data

EHR data were extracted sequentially within a 6-month window following the clinical evaluation to capture the complete diagnostic workflow at the Spine Center. Data included ICD-10 codes and imaging reports (e.g., MRI). EHR data were used to provide clinical context and describe the feasibility of linkage rather than to evaluate diagnostic accuracy or decision-making. ICD-10 diagnoses and imaging reports were extracted from routine clinical records and used descriptively to provide contextual information on diagnostic pathways and care trajectories. Reporting of analyses based on routinely collected EHR data followed principles outlined in the RECORD statement (10).

### Sample size and statistical analyses

The target sample size of ≥500 participants was chosen to ensure stable descriptive estimates and to support feasibility assessment and future profiling analysis (22). Descriptive statistics were used throughout, including means with standard deviations (SD) or medians with interquartile ranges (IQR) for continuous variables, and counts with percentages for categorical variables. No inferential analyses or subgroup comparisons were performed.

To contextualize the study population, baseline characteristics of included participants were compared descriptively with those of approximately 21,000 patients assessed at the Spine Center of Southern Denmark during the same recruitment period. These comparisons were intended to provide contextual representativeness rather than formal statistical inference.

### Interim feasibility assessment

After the first 100 participants, an interim feasibility assessment was conducted to evaluate recruitment efficiency, data completeness, and participant acceptability. Key metrics included average completion time for physical assessments, data completeness, incidence of adverse events or symptom exacerbations, and participant feedback collected via a brief post-assessment survey. No *a priori* numerical feasibility thresholds were defined, as the primary aim was to document real-world completion, burden, and safety to inform future protocol refinement rather than to evaluate success against predefined criteria. The resulting feasibility metrics are reported descriptively and may serve as empirical reference points for defining explicit feasibility thresholds in future confirmatory or implementation studies. Based on these findings, minor protocol refinements (such as more precise instructions and adjusted test sequencing) were implemented to enhance participant comfort and maintain data quality. No interim outcome analyses or hypothesis testing were performed.

## Results

Feasibility outcomes were the primary focus of the analysis and are therefore presented first, followed by descriptive summaries of patient-reported, performance-based, sensory, kinematic, and EHR-derived data.

**Figure 1:**
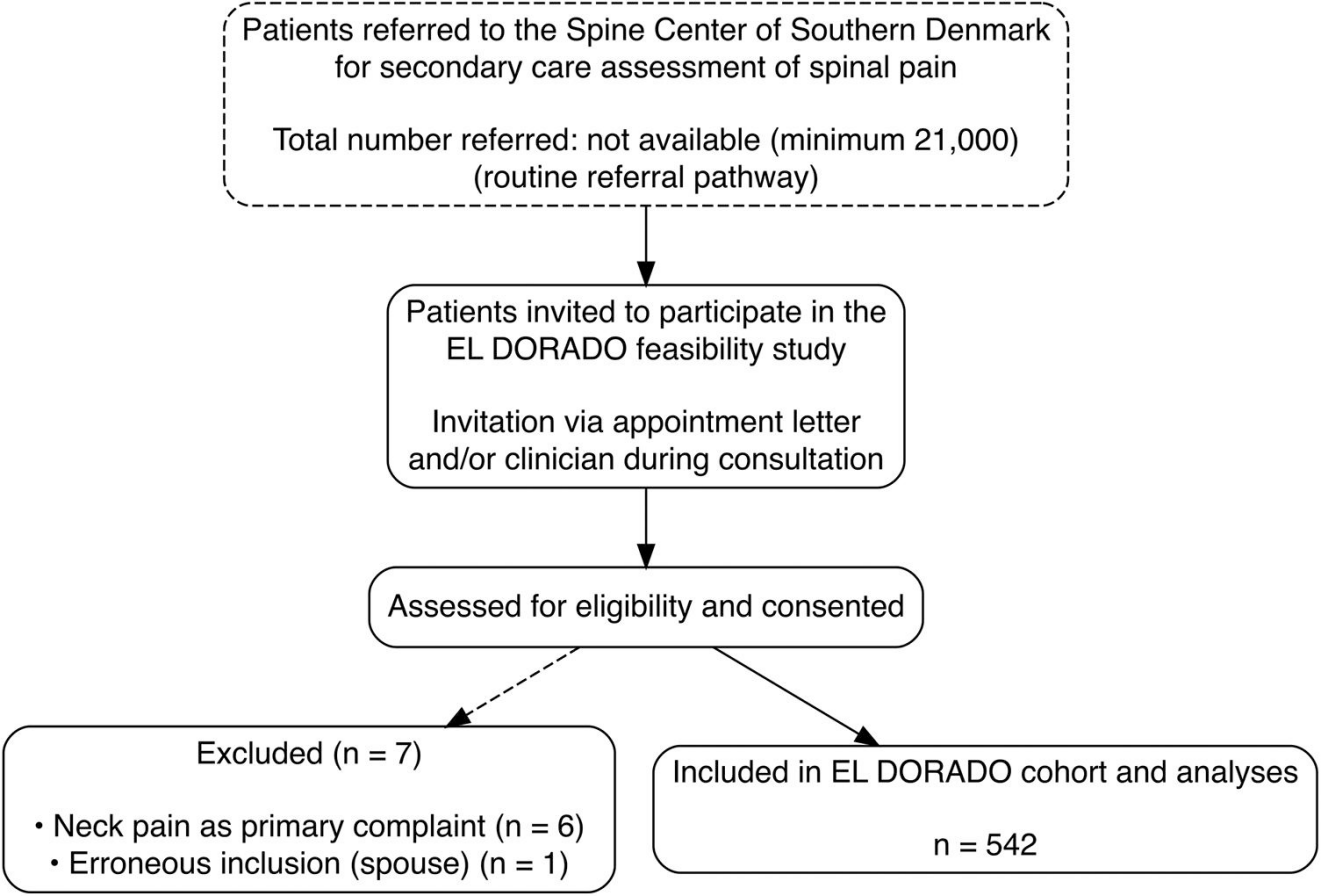
Participant recruitment and inclusion flow in the EL DORADO feasibility study. Flow diagram illustrating participant recruitment, exclusions, and final inclusion in the EL DORADO feasibility study. Participants were recruited through routine referral pathways to the Spine Center of Southern Denmark. The diagram shows the number of individuals initially included, the exclusions and their reasons, and the final analytical sample (*n* = 542). The figure is intended to document the feasibility and transparency of recruitment rather than representativeness or inclusion rates.

A total of 549 participants were initially included in the cohort. Seven individuals were excluded after enrollment (six with neck pain as the primary complaint and one individual inadvertently included as a spouse), resulting in a final analytical sample of 542 participants (Figure 1). Among the first 100 participants, the performance-based and kinematic components of the assessment battery were completed with a mean completion time of 22 min, and overall data completeness exceeded 95%. Participant acceptability was high, with 90% rating the assessment burden as minimal (data not shown). Only two minor adverse events occurred (one episode with loss of balance and one episode of dizziness), both of which resolved without sequelae.

Table 1 summarizes baseline characteristics selected *a priori* to contextualize the cohort relative to the broader Spine Center population. Overall, the cohorts were comparable in terms of age, sex distribution, body mass index, pain intensity, and disability. However, the EL DORADO cohort included a higher proportion of patients classified as high risk according to the STarT Back Screening Tool.

**Table 1:** Key baseline characteristics of the EL DORADO cohort compared with the spine center cohort.

Characteristic	EL DORADO (*n* = 542)	Spine center cohort	Completion (%)
Female sex, *n* (%)	278 (51%)	55.1%	100
Age, years (mean ± SD)	58 ± 15	56.6 ± 17	100
BMI, kg/m^2^ (mean ± SD)	27.8 ± 5.2	28.0 ± 5.8	91
Pain duration >1 year, *n* (%)	228 (45%)	43.5%	93
Low back pain intensity (0–10)	5.8 ± 2.2	6.0 ± 2.4	92
ODI score (0–100)	34 ± 15	35.2 ± 10.5	92
EQ-5D VAS (0–100)	55 ± 21	53.4 ± 22	92
STarT Back high risk, *n* (%)	260 (52%)	54,5%	92
Recent sick leave (<3 months), *n* (%)	135 (45%)	47.5%	55
MRI performed	479/542 (88.4)	-	100

Values are descriptive and selected *a priori* to contextualize the EL DORADO cohort. The Spine Center cohort reflects patients assessed during the same period; detailed diagnostic categories were not available at comparable granularity.

Patient-reported outcomes indicated moderate disability (mean ODI 34/100), poor health status (EQ-5D VAS 55/100), and a mean pain intensity of 5.8 for LBP and 4.9 for leg pain. Digital pain drawings were completed by 82% of participants. Functional capacity testing was completed by nearly all participants and demonstrated substantial impairments particularly for walking distance and sit-to-stand performance.

Markerless motion capture recordings were obtained for the majority of participants throughout the study period. During the early phase of data collection, recordings were successfully acquired, but pose estimation was not consistently possible due to ongoing calibration refinement. After finalization of the calibration setup (from participant 161 onward), pose estimation was successfully performed in 388 participants, with analyzable postural sway data available for 382 individuals (>98% of this subgroup). Lower overall completion rates reported across the full cohort, therefore, reflect technical maturation during early implementation rather than persistent limitations of the finalized protocol. Treadmill gait recordings showed substantially lower usability, primarily related to balance support requirements and walkpad-related tracking constraints.

Quantitative sensory testing was feasible in more than 95% of participants. At a descriptive, group level, the observed QST profiles were consistent with increased pain sensitivity, including lower pressure pain thresholds, enhanced temporal summation, short cold pressor tolerance, and limited conditioned pain modulation.

EHR linkage was achieved for all participants, providing diagnostic coding (69% non-specific LBP, 13% spinal stenosis, and 11% disc herniation) and imaging data, with MRI available in 88%.

Longitudinal follow-up via SMS was accepted by *317 participants (58%)*. Among those responding to at least one SMS (*n* = 300), the median (IQR) number of completed messages was 59 (13.25). Retention remained high, with 80% responding at 12 weeks, 79% at 24 weeks, and 75% at 60 weeks. Median weekly pain intensity remained stable at approximately 5/10, demonstrating the feasibility of long-term digital monitoring (Table 2). Detailed kinematic, postural sway, QST and SMS metrics are summarized descriptively in Table 2 and Supplementary Table S2.

**Table 2:** Feasibility metrics and selected functional and SMS outcomes.

Domain	Measure	Completion (%)	Summary value
Feasibility	Assessment battery duration	—	22 min (mean)
Feasibility	Overall data completeness	—	>95%
Feasibility	Minor adverse events	—	2 events
Functional capacity	2-Minute Walk Test	100	149 m (median)
Functional capacity	5x Sit-to-Stand	98	19.2 s (median)
Functional capacity	Hand grip strength	99	33.1 kg (median)
Follow-up feasibility	SMS retention at 60 weeks	75	Stable pain ∼5/10

Measures are presented descriptively to illustrate feasibility, burden, and cross-domain functioning. Detailed kinematic, sensory, and longitudinal metrics are provided in the Supplementary Material.

To illustrate potential concordance and discordance between self-reported disability and observed functioning across domains, exploratory descriptive comparisons were performed. These ODI-stratified summaries are presented for descriptive and illustrative purposes only. No formal trend testing, subgroup inference, or multidomain profiling was performed. Participants were stratified by ODI severity into established categories: 0%–20% (minimal disability), 21%–40% (moderate disability), 41%–60% (severe disability), and 61%–80% (crippled disability). Within each stratum, median values with IQR were summarized for selected performance-based outcomes and pressure pain thresholds (Table 3).

**Table 3:** Performance-based functional capacity and pressure pain thresholds stratified by Oswestry disability Index (ODI) severity.

ODI severity category/(*n*)	2-Minute walk test, m (median [IQR])	Hand grip strength, kg (median [IQR])	Sit-to-stand average time (s) (median [IQR])	PPT—tibialis anterior, kPa (median [IQR])	PPT—lumbar region, kPa (median [IQR])
0%–20% (minimal disability)/(103)	170 [46]	37.2 [16.5]	13.8 [7.2]	7,138 [1,621]	5,506 [4,306]
21%–40% (moderate disability)/(238)	152 [41]	32.2 [18.8]	17.5 [7.7]	6,998 [1,820]	4,663 [3,623]
41%–60% (severe disability)/(133)	128 [40.8]	32.7 [18.4]	19.1 [11.4]	6,616 [1,816]	3,893 [3,595]
61%–80% (crippled disability)/(26)	95 [47]	32.0 [28.6]	32.9 [22.4]	6,556 [1,085]	3,452 [4,268]
NA/(42)	144 [60.8]	35.5 [18.7]	17.4 [3.1]	7,078 [2,238]	5,957 [5,754]

Values are descriptive and intended to illustrate cross-domain concordance and discordance; no inferential statistical testing was performed.

Across increasing ODI severity categories, performance-based walking capacity demonstrated a graded decline, with the median 2-Minute Walk Test distance decreasing from 170 m in the minimal disability group to 95 m in the crippled disability group. Median hand-grip strength was modestly lower in the higher ODI severity categories, but with substantial overlap across strata. Pressure pain thresholds at both the tibialis anterior and lumbar region showed a progressive decrease with increasing ODI severity, indicating greater pain sensitivity at a descriptive level. Despite these trends, considerable overlap in functional and sensory measures was observed across ODI categories, illustrating cross-domain discordance whereby some individuals reporting moderate disability exhibited marked functional limitations, while others with high self-reported disability retained comparatively preserved performance.

## Discussion

This study demonstrates that a comprehensive, multidomain disability evaluation is both feasible and acceptable in a real-world hospital outpatient setting. We successfully enrolled 542 participants and achieved data completeness exceeding 95%. The test protocol was completed in approximately 22 min per participant, with no serious adverse events. Including collection of PROs via the SpineData registry (15–20 min), total participant time was typically 40–45 min. Staff time for setup, data recording, equipment calibration, and EHR extraction was approximately 15 min per participant, indicating that the workflow can be efficiently integrated into clinical routines. Participant feedback confirmed high acceptability, suggesting that multidomain assessment can be implemented in clinical practice without imposing excessive burden.

Across domains, we observed pronounced heterogeneity in functioning. Within the ICF framework, impairments at the body function level were reflected in altered pain processing and modulation (quantitative sensory testing, QST) and in psychological distress (PROs). Activity limitations were evident in performance-based tests, where participants demonstrated substantial reductions in functional capacity. The mean 2-Minute Walk Test distance (∼147 m) was approximately 20% below age-matched norms (23), and the mean Five-Repetition Sit-to-Stand time (∼22 s) was close to double that of healthy adults (24). Participation restrictions were evident in high rates of recent sick leave (45%) and poor health status (EQ-5D VAS 55/100). The exploratory cross-domain comparisons presented here are descriptive and illustrative and do not constitute formal multidomain profiling or subgroup identification. They illustrate how self-reported disability and observed functional capacity may diverge within a real-world hospital population. Such discordance was evident, with some participants reporting moderate disability yet exhibiting marked performance deficits, while others reported relatively low disability despite clear objective impairments. This pattern is consistent with previous evidence showing that self-report and performance-based measures capture distinct constructs and correlate only weakly to moderately in low back pain (9, 25, 26). Together, these findings underscore the limitations of single-domain evaluations and support the rationale for multidimensional assessment, while highlighting the need for future analytic work to formally integrate domains.

EHR data provided important contextual information, including MRI in 88% of participants and diagnostic coding for prevalent conditions such as spinal stenosis and disc herniation. These data inform chronicity, comorbidity, and care trajectories relevant to disability adjudication but remain susceptible to variability in coding and documentation (10, 27, 28). Standardized extraction and transparent reporting are therefore essential for consistent interpretation of EHR-derived information. The longitudinal SMS component further demonstrated the feasibility of remote follow-up, enabling differentiation between transient and persistent disability trajectories, which is relevant for understanding long-term symptom patterns rather than for informing benefit or rehabilitation decisions (29, 30). The observed pain trajectories appeared less favorable than those reported in some previous studies (31), suggesting that the cohort represents a population with relatively high risk profiles; however, no prognostic inferences are drawn from these descriptive data.

This multidomain design exemplifies the multidimensional model of functioning articulated in the ICF. Each domain contributed unique information: QST captured sensory and pain-modulatory impairments; performance tests quantified activity-level limitations; PROs represented perceived participation and psychosocial factors; and EHR data situated these findings within diagnostic and clinical contexts. These considerations align with recent calls for multidimensional, ICF-consistent assessment frameworks in rehabilitation (20, 32).

In principle, multidomain assessment infrastructures may, if supported by future empirical work, contribute to more transparent and potentially equitable approaches to disability evaluation. Importantly, no administrative, compensation, or benefit-related decisions were informed by the data collected in this study. However, equity-related outcomes, misclassification, and clinical or administrative decision-making were not empirically assessed in this feasibility study, and no conclusions can be drawn regarding such effects. All applied implications discussed here should therefore be regarded as hypothesis-generating rather than evidence of demonstrated benefit.

Key strengths of this study include the large, real-world cohort, comprehensive coverage of ICF domains, and high data completeness. Limitations include the single-center design and descriptive nature of the analysis, which preclude conclusions on predictive validity or clinical outcomes. The referral-based recruitment and absence of a known eligible denominator limit inferences about representativeness and generalizability. Patients who declined to participate may differ from included participants in symptom severity, functional capacity, or psychosocial burden, which could influence the external validity of the findings. The cohort likely reflects a population with more complex or persistent LBP than primary care populations. Early kinematic data suffered from tracking losses, though protocol refinements markedly improved recording quality. Although pose estimation was successfully achieved for most participants after calibration finalization, kinematic outcomes were not analyzed in this study and should be regarded as infrastructural rather than analytically mature. Therefore, they were not interpreted as clinically meaningful or inferential in the present analyses without further validation. Furthermore, performance-based outcomes may be influenced by motivation or pain behavior, necessitating further validation of ecological validity. A software error resulted in missing data for one QST variable (temporal summation after the cold pressor test) in 103 participants, though this did not materially affect feasibility conclusions.

## Conclusion

This study demonstrates that a comprehensive, multidomain assessment of functioning in patients with LBP can be feasibly integrated into hospital outpatient care, with high data completeness, acceptable time burden, and no serious adverse events.

While the present study integrates multiple complementary domains of functioning, it does not perform formal multidomain profiling or evaluate clinical or administrative decision-making. Instead, the findings illustrate how different domains may provide concordant or discordant perspectives on functioning in a population with complex and persistent LBP. These descriptive observations support the rationale for multidimensional assessment but should not be interpreted as evidence of improved diagnostic accuracy, prognostic performance, or equity.

By establishing a harmonized dataset spanning patient-reported, performance-based, sensory, kinematic, and clinical record domains, this study provides an infrastructure for future research on prognosis, subgroup identification, and disability evaluation. Further research is needed to determine whether multidomain approaches enhance clinical decision-making, administrative disability assessment, or equity across patient subgroups.

## Data Availability

The raw data supporting the conclusions of this article will be made available by the authors, without undue reservation.
