# Supplementary material for: Integrated disability evaluation in low back pain: feasibility and multidomain profiling from the EL DORADO cohort
Source: Front Rehabil Sci. 2026 Mar 18;7:1731260. doi: 10.3389/fresc.2026.1731260 (PMC13038888; doi:10.3389/fresc.2026.1731260)
Supplement: Supplementary file 1 [file Datasheet1.docx]

# Supplementary Tables

## Supplementary Table S1.

## Completion rates for markerless kinematic assessments

| Kinematic task | Completion (n/N, %) |
| --- | --- |
| Postural sway | 382/542 (70.3%) |
| Sit-to-stand (kinematic recording) | 381/542 (70.3%) |
| Spinal flexion–extension | 380/542 (70.1%) |
| Spinal lateral flexion | 379/542 (69.9%) |
| Pencil pick-up | 379/542 (69.9%) |
| Lifting task (6 kg) | 368/542 (67.9%) |
| Treadmill gait – unaided | 257/542 (47.4%) |
| Treadmill gait – with bar handle support | 196/542 (36.2%) |

Completion reflects technically valid recordings available for post-processing. Lower completion rates for treadmill gait primarily reflect balance support use and tracking constraints in a routine clinical workflow.

## Supplementary Table S2.

## Detailed quantitative sensory testing (QST) outcomes

| QST measure | Completion (n/N, %) | Median (IQR) | Unit / Notes |
| --- | --- | --- | --- |
| Pressure pain threshold – lumbar spine | 541/542 (99.8%) | 4785 (4272) | kg |
| Pressure pain threshold – tibialis anterior | 542/542 (100.0%) | 3968 (3603) | kg |
| Temporal summation (ΔVAS) – tibialis anterior | 542/542 (100.0%) | 7.0 (24) | VAS |
| Cold pressor tolerance time | 538/542 (99.3%) | 30.5 (51.8) | seconds |
| Cold pressor maximum pain | 538/542 (99.3%) | 90 (20) | VAS |
| Cold pressor pain AUC | 538/542 (99.3%) | 2140 (3860) | VAS·s |
| Conditioned pain modulation – ΔPPT | 541/542 (99.8%) | 0.0 (11) | kg |
| Conditioned pain modulation – ΔTS | 438/542 (80.8%) | 1.0 (16) | VAS |

Temporal summation after the cold pressor (used for CPM ΔTS) was missing in 103 participants due to a software error during early data collection.

## Supplementary Table S3.

## SMS follow-up feasibility and retention

| Follow-up metric | Completion (n/N, %) | Mean (SD) | Median (IQR) |
| --- | --- | --- | --- |
| Answered first SMS (week 1) | 283/317 (90.1%) | 6.6 (2.4) | 7 (3) |
| Retention at 12 weeks | 252/317 (80.3%) | 5.9 (2.4) | 6 (4) |
| Retention at 24 weeks | 248/317 (79.0%) | 5.7 (2.6) | 5 (5) |
| Retention at 60 weeks | 235/317 (74.8%) | 5.4 (2.7) | 5 (4) |

SMS data are reported descriptively to document feasibility and retention of long-term digital follow-up.
